# Biological Acoustic Levitation and Its Potential Application for Microgravity Study

**DOI:** 10.3390/bioengineering12050458

**Published:** 2025-04-25

**Authors:** Taylor Boudreaux, Luke Freyhof, Brandon D. Riehl, Eunju Kim, Ryan M. Pedrigi, Jung Yul Lim

**Affiliations:** 1 Department of Mechanical and Materials Engineering, University of Nebraska-Lincoln, Lincoln, NE 68588, USA; tboudreaux2@huskers.unl.edu (T.B.); briehl4@unl.edu (B.D.R.); nesprin1747@gmail.com (E.K.); rpedrigi@unl.edu (R.M.P.); 2 Department of Biological Systems Engineering, University of Nebraska-Lincoln, Lincoln, NE 68588, USA; lfreyhof2@huskers.unl.edu; 3 Nebraska Center for Integrated Biomolecular Communication, University of Nebraska-Lincoln, Lincoln, NE 68588, USA; 4 Nebraska Center for the Prevention of Obesity Diseases, University of Nebraska-Lincoln, Lincoln, NE 68588, USA

**Keywords:** acoustic levitation, microgravity, clinostat, open and contactless environment, biological application of acoustic levitator

## Abstract

The open and contactless environment of acoustic levitation provides a unique condition in experimenting with varying substances while levitated for observation and implementation with other devices, with recent improvements in cost and accessibility. We briefly decipher the theory behind acoustic levitation and describe currently available levitation platforms. Then, how these platforms have been employed in biological applications is reviewed. Intriguingly, recent researches indicated the viability of acoustic levitation to be utilized as a microgravity simulator. We introduce existing on-ground microgravity platforms, and discuss the potential of acoustic levitation in simulating microgravity. Acoustic levitation could be an alternative to microgravity platforms such as clinostats while allowing for novel microgravity research. On the other hand, the microgravity provided by acoustic levitation may be restricted due to potential limitations in the available levitation volume, relatively larger gravity compared to 10^−3^ g centrifugal acceleration from clinostats, and probable instability due to air perturbations and acoustic streaming. With more knowledge about in-droplet particle rotation and the regulatory factors during levitation, acoustic levitation may provide a new and advanced platform for microgravity simulation via taking advantage of its availability for real-time observation and manipulation of samples via added instrumentation while samples are levitated in a simulated microgravity condition.

## 1. Introduction

The levitation of substance without physical contact—one of the most intriguing physical phenomena—gathers significant interest in scientific communities. A variety of levitation methods have been developed including levitation via magnetic, aerodynamic, optical, electrostatic, and acoustic tools. Each of these methods employs a balancing force that acts on the substance through the surrounding medium without physical contact, rendering the substance suspended in the medium (mostly air) [1]. Acoustic levitation—the topic of this review—balances gravity with an acoustic radiation force (ARF) wherein substance is levitated on the nodes of a standing ultrasonic wave [2]. One of the benefits of acoustic levitation is its ability to be used universally without being limited by the material properties of the levitation target, in comparison with the case of diamagnetic levitation which requires the levitated sample to be at least weakly diamagnetic [3]. Moreover, recent innovations in acoustic levitation platforms have provided an affordable and accessible option for small-scale levitation experiments [4].

One intriguing application of acoustic levitation that has been proposed more recently is microgravity simulation [5,6,7]. As humanity continues to look beyond the earth, the demand for a greater understanding of the effects of space travel and living long term under microgravity increases. Living organisms have evolved and lived under a constant 1 g acceleration. When this is taken away in a microgravity environment, significant biological impacts may occur such as loss of bone density [8], loss of cardiac function [9], and muscular atrophy [10]. High costs and limited accessibility of spaceflight and limited experimental times of other in-flight microgravity observation methods such as parabolic flights and drop towers have led to a strong need for on-ground microgravity simulation platforms. The commonly used on-ground microgravity platforms utilize rotation motion to average the gravity vector applied to a sample to zero. Such a microgravity simulating method has been attempted in a clinostat, random positioning machine (RPM), and rotating wall vessels (RWV). On the other hand, acoustic levitation has not yet been intensively implemented for the same purpose of microgravity simulation, while its potential to mimic microgravity has been proposed [5,6,7].

This review gives an overview of the acoustic levitation technology and its current applications in different research focused on biological effects. Then, existing on-ground microgravity simulation platforms are introduced. Finally, a potential application of the acoustic levitation technology in microgravity research will be discussed with regards to feasibility and future directions.

## 2. Acoustic Levitation

### 2.1. History of Acoustic Levitation

The effects of acoustic forces on particles have been observed since at least 1866 when the motion of dust particles in resonant tubes was first studied [11]. Then, the behaviors of both compressible and incompressible spheres in an acoustic field were observed [12,13]. In 1961, Gor’kov created a simplified model for determining forces acting on a particle in an arbitrary acoustic field, in which the forces acting on particles much smaller than the acoustic wavelength could be modeled [14]. In 1967, a similar model was created by Nyborg that could also be utilized to approximate the acoustic forces on a levitating particle [15].

### 2.2. Concept of Acoustic Levitation

**Figure 1:**
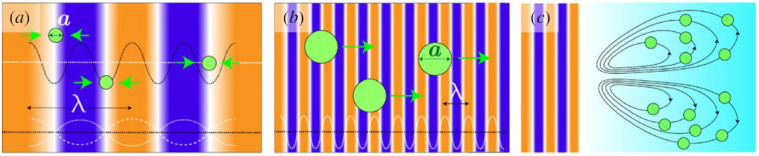
Acoustic radiation forces acting on particles (green dots) in an acoustic field as determined by the particle size (a) and wavelength (*λ*) of the soundwave. (**a**) When ka≪1, the gradient force acts on the particles and they are trapped in the low-pressure nodes. (**b**) When ka>1, the scattering force acts on the particles and they are moved along the direction of the wave. (**c**) When ka<1, acoustic streaming can result in drag forces that act on the particles [16].

Particles can be acoustically manipulated in a medium via multiple forces: gradient, scattered, and streaming forces [16]. The gradient and scattered forces comprise the acoustic radiation force described by the Gor’kov model. The gradient force pushes the levitated particle into a low potential or low-pressure zone surrounded by high-pressure zones (Figure 1a). The scattered force pushes the levitated object along the flow direction of the acoustic waves (Figure 1b). The streaming-induced force (Figure 1c) results from fluid movement due to acoustic streaming, a phenomenon that can occur when an acoustic field is present in the fluid. This fluid movement can push on an object and balance out the force of gravity acting on that object. In the models that describe these forces, levitation is strongly dependent on the largest dimensional size of the particle [17,18,19]. The concept is that the balance between the gradient and scattering forces is determined by the factor, *ka* (where *k* = 2*π*/*λ*, *λ* is the wavelength of soundwave, and *a* is the largest particle size):

(1) $$ka=\frac{2\pi \left(Particle\;size\right)}{\lambda }$$

When the levitated particle is much smaller than the acoustic wavelength, then the gradient forces dominate. On the other hand, if the particle being levitated is much larger than the acoustic wavelength, the scattering forces dominate. See the review by Mohanty et al. [16] for a more in-depth analysis of these forces involved in acoustic levitation.

The Gor’kov model is limited to describing the acoustic radiation force for spherical objects that are much smaller than the acoustic wavelength (or Rayleigh scattering regime) [20]. The Gor’kov model also ignores acoustic streaming, acoustic viscous torques, and harmonic generation [21,22].

Recent advancements in non-resonant levitators such as TinyLev utilize standing waves and have the ability to manipulate wave node locations [4]. These node locations can be changed by altering the phases of the opposing acoustic waves generated. Changing the voltage signals sent to the transducers also causes a change in the pressure potential present in the medium such as air. This allows for the manipulation of a particle levitating in a 3D situation.

It is notable that acoustic levitation may have some limitations. First, a medium is required for acoustic manipulation. This medium, most often air, is required for the acoustic waves to propagate, and thus, acoustic levitation cannot be applied in a vacuum. Additionally, the mass and size of a particle can limit its ability to levitate [23]. Particle rotational control needs to be further developed before 3D bulk acoustic wave levitation can be used for research that requires precise control over particle rotation and orientation [24,25]. Acoustic levitation can also be disturbed by the movement of the surrounding medium. For example, if there are air drafts present in the area surrounding an acoustic levitator, the rotation of a levitated particle can be altered, or the particle can even be pushed out of the trapping field.

### 2.3. Acoustic Levitation Platforms

**Figure 2:**
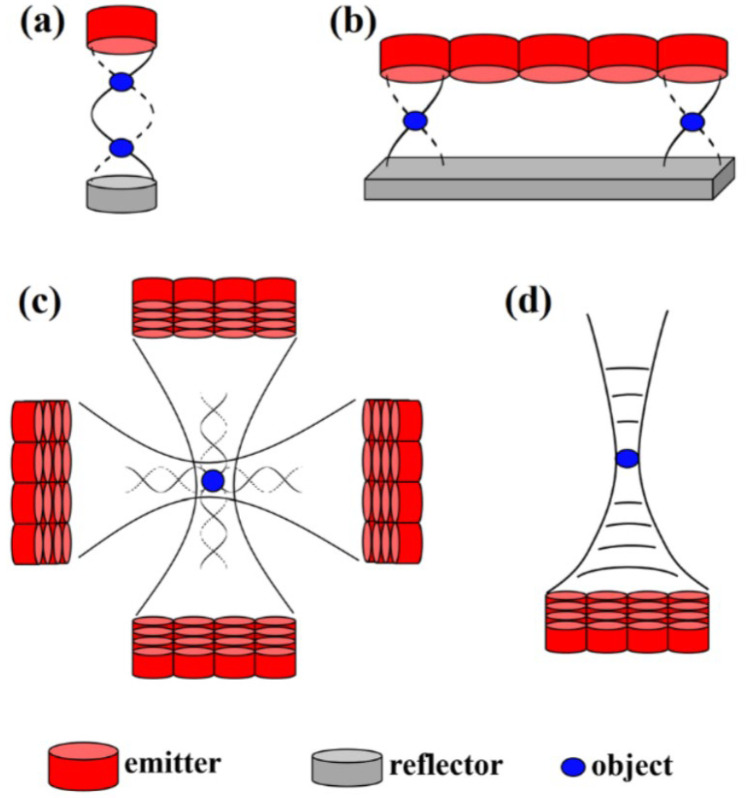
Various acoustic levitation setups. (**a**) Single-axis. (**b**) Two-dimensional Langevin-type transducer array with reflector. (**c**) Four perpendicular arrays of ultrasonic emitters. (**d**) Single-beam trapping [20].

Acoustic levitation platforms utilize ultrasonic transducers to apply acoustic radiation force to a sample. There are several different forms that acoustic levitators can take (Figure 2). Three major categories that these levitators can fit in are single-beam, resonant, and non-resonant trapping. The simplest platform is single-beam trapping, which uses an upward facing ultrasonic transducer to balance the gravitational force with acoustic radiation force to levitate the sample.

**Figure 3:**
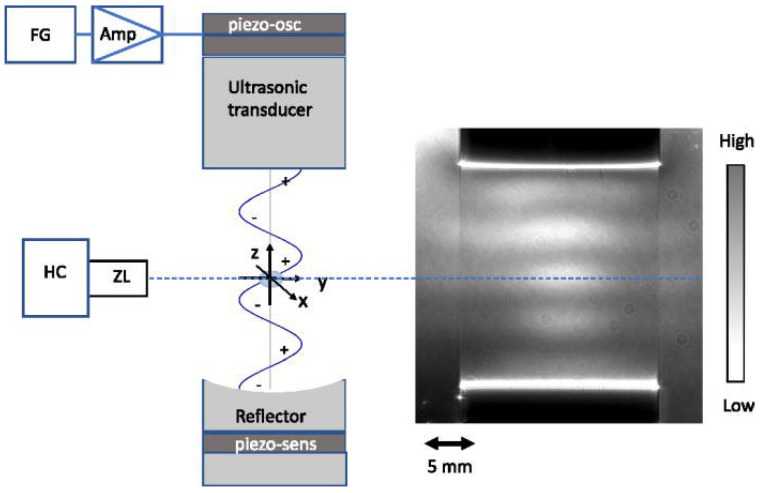
Schematic and imaging of pressure distribution in a Langevin-type single-axis acoustic levitator. The input from a function generator (FG) is magnified by the amplifier (AMP) and fed into a piezo-oscillator, exciting the ultrasonic transducer. The ultrasonic wave is reflected by a mirror reflector connected to a piezo-sensor. HC: high-speed camera; ZL: zoom lens. The image on the right examples time-averaged pressure distribution captured by HC [29].

Resonant levitators use concave reflectors to produce a stronger trapping force than a single-beam setup. These platforms can support higher trapping forces because they are able to produce higher voltage input signals [26]. Resonant levitators function by creating a pressure potential field using the geometry of a wave produced by a generator/transducer and its reflection. Samples placed in the levitator can affect the field resonance and thus change the trapping force. These levitators are sensitive to air conditions such as temperature and humidity [27]. Resonant levitators are also sensitive to changes in geometry of both the equipment and the presence of a sample [28]. One of the most common resonant levitation platforms is the Langevin horn. This is a horn-shaped resonator coupled with a piezoelectric ultrasonic transducer (Figure 3) [29].

A limitation to Langevin horns is that they are difficult to tune and require high voltages which can be dangerous if the operator is exposed to the circuit. They can also heat up throughout use due to these high voltages, which can lead to changes in the acoustic field and heating of the levitated sample [30,31]. Langevin horns therefore require time to warm up before being able to produce a stable acoustic field, and temperature-dependent properties of the levitated sample need to be considered.

**Figure 4:**
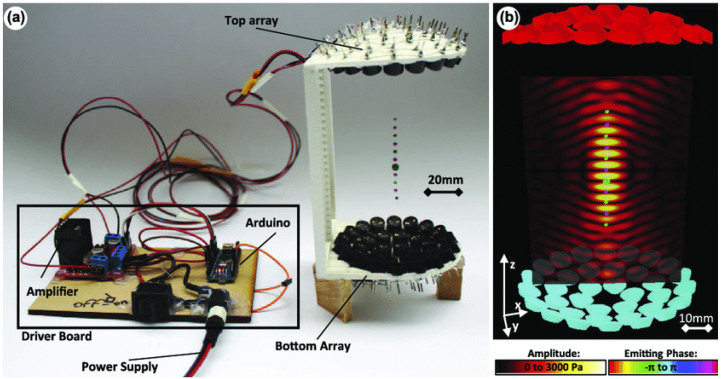
Overview of the TinyLev acoustic levitation platform. (**a**) TinyLev consists of a single-axis levitator with two 36 transducer arrays which are controlled by a driver board. (**b**) Modeled acoustic field showing the emitting phase of the transducers and the pressure at a given point in the field [4].

Non-resonant levitators function by creating a pressure potential field using the intersecting wave geometry of two waves created by two opposing sets of transducers [32]. The phase delays can be created either through physical geometry arrangements (changes in the vertical height of the transducers), or electronically through offsets in the signals used to generate the acoustic waves [4]. Levitated particles can be manipulated by changing the phase of the waves produced by one of the transducers. This alters the locations of the pressure nodes, thereby changing where the particle levitates. Recently, Marzo et al. [4] developed the TinyLev (Figure 4) device, which has made non-resonant levitation a much more affordable and accessible alternative. One limitation of TinyLev is that it is not quite as powerful yet and cannot generate as high acoustic trapping forces. Moreover, TinyLev is still being proven in its abilities to levitate a wide variety of materials.

### 2.4. Acoustic Levitation of Biological Samples

**Figure 5:**
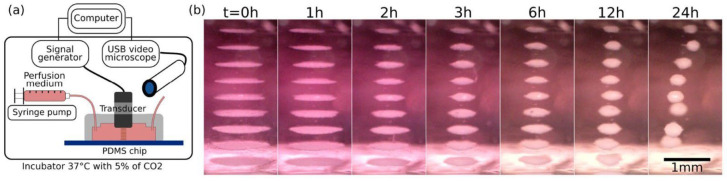
MSC cell sheets self-organize into spheroids when exposed to an acoustic field. (**a**) Model of the experimental setup where an ultrasonic transducer is used on the cell medium and recorded with a video microscope. (**b**) Over time, MSC cell sheets orient into spheroids [33].

**Figure 6:**
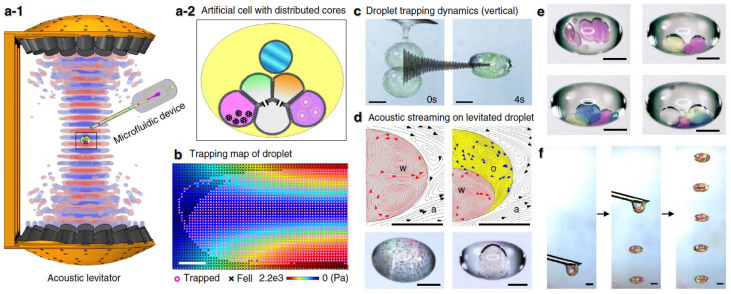
TinyLev-based droplet lab. (**a-1**) Model of TinyLev platform wherein the microfluidic device can be placed. (**a-2**) Schematic showing the artificial cell with distributed cores. (**b**) Map showing the simulation results of acoustic trapping in air (2.2e3 Pa = 2200 Pa). (**c**) The droplet oscillates until it stabilizes when trapped. (**d**) Simulated (**top**) and experimental (**bottom**) streaming in a droplet of water (**left**) and both water and oil (**right**). (**e**) Water droplets manually deposited inside levitated oil droplets. (**f**) Multiple artificial cells placed in low-pressure nodes [25].

There have been studies that explore the biocompatibility and applications in biological samples of acoustic levitation [5,6,25,33,34,35]. Jeger-Madiot et al. [33] explored the use of acoustic levitation for the self-organization of cell cultures into spheroids. Spheroids are 3D cell assemblies that have shown improved physiological relevance to in vivo cellular conditions compared with typical 2D layered cultures. The main factor limiting the implementation of spheroids into clinical settings is the difficulty in the predictable reproducibility of spheroids [36]. Jeger-Madiot et al. [33] placed sheets of mesenchymal stem cells (MSCs) into the acoustic levitation field, and allowed the cultures to levitate. They achieved spheroid formation after a characteristic period of 10 h levitation (Figure 5). They further showed that MSCs remained viable post levitation. Wang et al. [34] demonstrated the ability of ultrasonic manipulation to build reconfigurable arrays of protocells and natural cells. Utilizing this, they evidenced chemical signal transduction within the cellular network via a two-step enzymatic cascade reaction. Intriguingly, Li et al. [25] further extended this potential capability by demonstrating that acoustic levitation could be performed in an open, container-less environment (Figure 6). They developed an artificial cell and manipulated it using acoustic levitation and other associated methods implemented during levitation to study the physical and biochemical effects. The open environment also provided a significant amount of space, allowing for other instruments to be used. Via this approach, they could successfully construct the droplet networks of cells using associated microfluidics and rearrange the cells using acoustic manipulation. This study strongly supports the use of acoustic levitation to effectively manipulate cell networks and phenotypes with additional capability to implement other instruments to be associated.

Tsujino and Tomizaki [37] applied an acoustic levitator for protein crystallography. They combined the acoustic levitator with a high frame rate pixel array detector for X-ray crystallography. They applied this equipment to assess protein crystals inside liquid droplets. The protein crystals rotated quickly inside the droplets during levitation, which enabled the sampling of a reciprocal space in an efficient manner. They then processed the collected data using serial femtosecond crystallography (SFX) and modeled the protein structure using molecular replacement. Based on the electron density and temperature factor distribution, they demonstrated that the crystals were not damaged during levitation supporting the viability of the method. They concluded that acoustic levitation could increase efficiency and reduce the amount of handling required for protein crystallography, suggesting an important step toward complete automation.

Kepa et al. [35] built on the crystallography capabilities demonstrated by Tsujino and Tomizaki [37], and observed thin films that are acoustically levitated for protein crystallography. They emphasized the application of acoustic levitation as a container-less environment that can be used across any material, unlike magnetic levitation. They created thin films to provide a surface for protein crystallography and engineered the shape to minimize rotation to 1–4 rpm. These films allowed high-viscosity samples to be more easily placed in the levitator. The open environment of the setup allowed for the implementation of X-ray diffraction testing. Via this approach, viscous proteins such as those in the liquid-cubic phase (LCP) could be loaded in the acoustic field and X-ray crystallography could be performed. The ability to perform X-ray crystallography on LCP proteins is important for discovering structures that can be used in pharmaceutical production [38].

## 3. Current On-Ground Microgravity Simulation Platforms

There have been significant advances in on-ground microgravity simulation platforms. Before discussing the potential usage of acoustic levitation for microgravity, we will briefly review current microgravity simulators. For more information, see the papers by Kiss et al. [39] and Ferranti et al. [40].

The way to obtain a real, long-term microgravity environment is through spaceflight. However, the high cost and limited access to spaceflight prevent this from being a viable option. Less expensive and more accessible options are thus required that can provide a weightless environment. Drop towers, parabolic flights, and sounding rockets have been used to provide microgravity environments. Drop towers provide the most comparable microgravity conditions to spaceflight while being the cheapest of these alternatives. However, the experiment time of drop towers is limited to 5–9 s [41]. Sounding rockets can also generate high-quality microgravity conditions from 10^−2^ to 10^−4^ g, but the costs can be similar to those of satellites and the International Space Station (ISS). The experiment times of sounding rockets are still limited to around 4–13 min [41].

**Figure 7:**
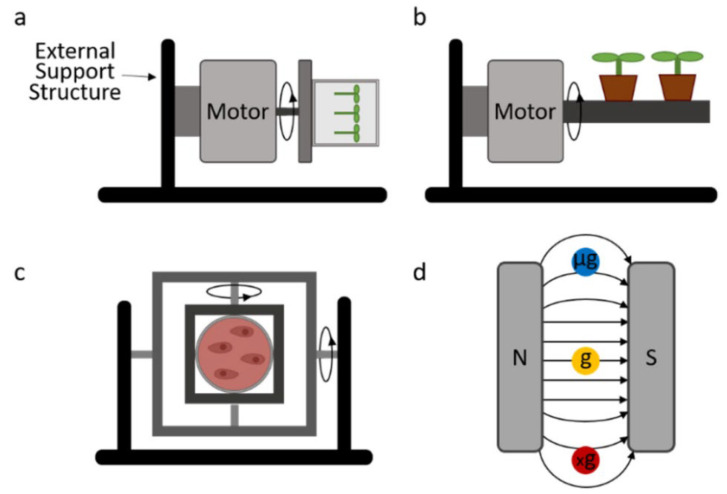
Common ground-based microgravity simulators. (**a**) One-dimensional clinostat. (**b**) Two-dimensional clinostat. (**c**) Three-dimensional clinostat/RPM. (**d**) Diamagnetic levitation [40].

Due to the limiting factors of these methods, many on-ground microgravity simulators have been developed (Figure 7). Rather than providing an environment where there is reduced gravity, these devices aim to counteract gravity. This is commonly carried out in one of two ways: by reducing the experienced gravity vector to zero through rotational methods, or by counterbalancing gravity with an electromagnetic force (EMF).

### 3.1. Clinostat

**Figure 8:**
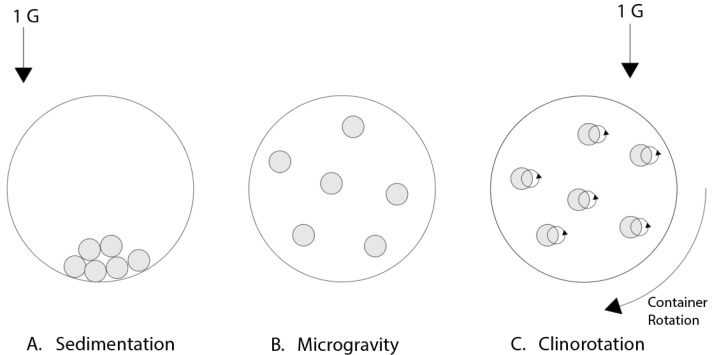
Demonstration of how a fast-rotating clinostat simulates microgravity. Without rotation, there is sedimentation of the particles due to gravity (**A**). In microgravity, there is uniform distribution throughout the container due to a lack of gravitational force (**B**). With clinostat rotation, the particles move in a circular pattern (**C**) (note: the particle rotation observed in C is relative to the container). As the speed of rotation increases to a certain point, the radius decreases until the particle is nearly static, similar to microgravity. Adapted and redrawn from [48].

The simplest microgravity simulator is the clinostat, which involves the rotation of a sample around one or two axes. A 2D clinostat rotates the sample around a single horizontal axis [42,43]. While there are several ways a clinostat can be set up for different applications, a shared mechanism of all clinostats is a constant rate of rotation. This is applied for both 2D and 3D clinostats, and distinguishes a 3D clinostat from an RPM. Low-speed clinostats rotating at speeds around 2–4 rpm are often used for larger samples such as whole plants. High-speed clinostats, which generally rotate at 30–150 rpm, can be used for cell samples since the increased rotation rate prevents sedimentation of the particles in the sample, keeping them suspended in the medium [44]. Clinostats operate based on the principle of clinorotation [45,46,47]. By rotating the sample at a constant rate, the gravity vector is averaged to zero, effectively eliminating the effects of gravity to target around 10^−3^ g centrifugal acceleration. For fast-rotating clinostats, when rotated at the proper speed, the particles in a liquid suspension will stabilize and the rotation radius will move to zero, replicating a microgravity environment (Figure 8) [48]. Clinostats are the cheapest of all microgravity simulation platforms. They are simple to create and control, particularly for 2D clinostats which only require constant rotation about a single axis. Clinostats have been widely used and modified such as combining with potential real-time observation. One thing to note is that as with all rotational platforms, mechanical stresses such as fluid shear are inevitable. The 2D clinostats have been used in cellular microgravity research, for example, recent reports on the effects of clinostat microgravity on the nuclear localization of a key mechanosensitive transcription regulator, yes-associated protein (YAP) [49,50,51]. The findings supported the result on YAP from ISS experiments [52] that implemented downregulated YAP activation under the microgravity condition.

### 3.2. Random Positioning Machine (RPM)

The setup of the RPM device is analogous to the 3D clinostat. However, unlike the 3D clinostat providing 3D constant rotation [53,54], the rotation rate in each gimbal frame of the RPM is constantly changing to provide random sample orientation. Using specialized algorithms, the rotation rates of each frame are randomly varied at predetermined intervals to maintain the net gravity vector near zero [55]. The rotation rate of the RPM needs to be maintained within a range that fulfills two criteria [56,57]: the rotation should be fast enough to prevent a sample from being able to adapt to the gravity vector but slow enough to prevent significant centrifugal acceleration on the sample. Using an RPM, high-quality microgravity conditions can be obtained, e.g., up to around 10^−4^ g [58]. The RPM has proven to be an effective analog to microgravity for some studies, providing results more comparable to real microgravity compared to clinostat. Using an RPM, the central columella cells of Arabidopsis root tips, which are known to be gravity-perceiving cells, were studied [59]. They compared the plastid position of cells grown on the ground, in spaceflight, in the RPM, and in a clinostat. They found no statistical difference in plastid position between spaceflight and the RPM, but they did find a significant difference between spaceflight and samples subjected to a clinostat. On the other hand, the result may vary with the biosystem under investigation. In a study examining Chara rhizoids, Krause et al. [60] concluded that a fast 2D clinostat produced results more representative of microgravity. They reported that the microgravity condition with an RPM was not improved over a 2D clinostat but resulted in greater dispersion due to increased vibrations. It is notable that results can be affected by the mechanical forces present in each rotational platform, and the additional axis and increased rotation complexity can make an RPM difficult to control [60]. Additionally, the changes in direction can generate accelerations which may affect the accuracy of results. Another limiting factor of an RPM is the small effective sample size. Due to the two axes of rotation, the position of an optimal microgravity condition where the two axes intersect is small. Moreover, as a sample moves from the center, the centrifugal acceleration will increase, thereby decreasing the quality of microgravity.

### 3.3. Rotating Wall Vessels (RWVs)

**Figure 9:**
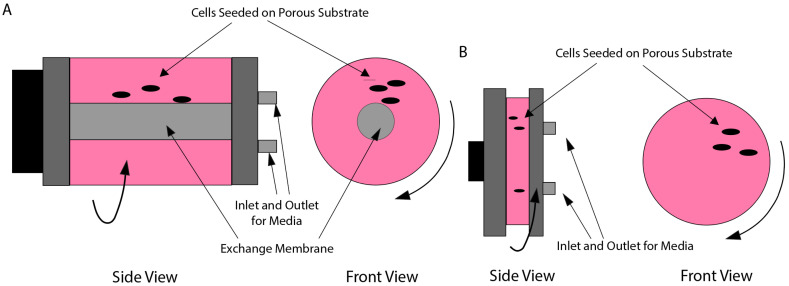
Varieties of RWV bioreactors. (**A**) Slow-turning lateral vessel (STLV). (**B**) High-aspect ratio vessel (HARV). Adapted and redrawn from [61].

RWVs operate with the same rotational principles as a 2D clinostat; however, they allow for a larger sample volume (Figure 9) [61]. The RWV was originally developed by NASA [62] to protect cell cultures on space shuttle missions during takeoff and landing but has found use in microgravity simulation. An RWV rotates around 10–20 rpm, which is determined such that the rotational frequency matches the sedimentation velocity to keep the cells suspended. One of the primary benefits of an RWV is the increased sample size, which improves the capability of statistical assessment of the data. Additionally, an RWV-based bioreactor can have membranes in the vessel used for oxygen exchange, allowing for more comprehensive biological experiments [63]. This setup also allows for experimentation with spheroids that can provide more realistic representations of in vivo cellular conditions. Furthermore, the low shear stress and high mass transfer provided by the RWV bioreactor are suitable for studies on cell behavior in a more physiological environment to target cell differentiation, metabolism, and 3D tissue culture [64,65,66]. A limiting factor of RWVs on the other hand is that high-density cell cultures will deposit, so culture densities need to be limited to prevent sedimentation.

### 3.4. Diamagnetic Levitation

Diamagnetic levitation is used to simulate microgravity in a different way than the previous methods. Instead of averaging the gravity vector to zero using rotation, diamagnetic levitation provides a microgravity condition by balancing gravity with a magnetic force [67]. This balancing occurs at a molecular level in biological samples due to the diamagnetic nature of cells. There are several types of magnets used for diamagnetic levitation. By balancing at a molecular level, diamagnetic levitation does not subject samples to the same mechanical stresses experienced by rotational platforms as described above. Additionally, because a magnetic field can produce a gradient, partial, or hypo-gravity condition that can be simulated based on the sample’s location in the field, tuning the technique for the replication of specific gravitational effects is required [68]. Moreover, the strong magnetic field has been shown to have significant impacts on sample organization and function [69]. Valiron et al. [70] specifically demonstrated that neurons may lose cellular organization when exposed to a strong magnetic field. This may limit the applications of diamagnetic levitation as a microgravity simulator for biological samples.

## 4. Acoustic Levitation as a Microgravity Simulation Platform

While relatively little research currently exists about the microgravity conditions produced by acoustic levitation, studies have proposed its viable potential. For example, Li et al. [5] and Cao et al. [6] described the probable usage of acoustic levitation as a tool to provide microgravity. Recent reports by Vashi et al. [7,71] provided a theoretical basis for acoustic levitation to be utilized as a functional microgravity simulator and proposed space-mimicking applications.

Li et al. [5] examined the acoustic levitation of zebrafish embryos and assessed their biocompatibility. It was found that there existed slight toxicity when embryos were exposed to acoustic levitation, which was ameliorated with the growth of embryos. For zygote-phase zebrafish that were levitated 1 h after fertilization, an 82% mortality rate was observed. The zebrafish levitated 32 h after fertilization, however, displayed a 0% mortality rate. They further proposed that there exists a microgravity environment in the formed levitation droplet. They claimed that the status of zebrafish being in a suspended situation is analogous to microgravity as zebrafish in their system do not perceive the earth’s gravity. They also commented on the realistic benefits of acoustic levitation that could separate it from other methods in the case where it can be defined as a microgravity simulator. For instance, the contactless environment that allows for the transport of materials has the potential to be used for drug delivery, diagnosis, and artificial insemination. The potential of drug loading via acoustic levitation was also proposed in a report by Benmore and Weber [72].

Cao et al. [6] explored the use of acoustic levitation for crystallization, and proposed that ultrasonic levitation could find use as an on-ground microgravity simulator. Specifically, they assessed that the single-axis acoustic levitator stably levitating a droplet inside a levitation chamber can function as a valid on-ground microgravity simulator. On the other hand, they mentioned the potential limitation. They described that acoustic streaming, non-uniformity in the field, and the convection phenomenon in acoustic levitation may result in uncontrollable rotation and mass transfer beyond the microgravity condition, thereby requiring solutions to mitigate these effects. Acoustic streaming can potentially impact biological samples by generating fluid flows that impose shear stress, alter mass transfer, and thereby influence cell behavior. These effects could result in increased cell membrane permeability, altered proliferation, and migration [73,74].

**Figure 10:**
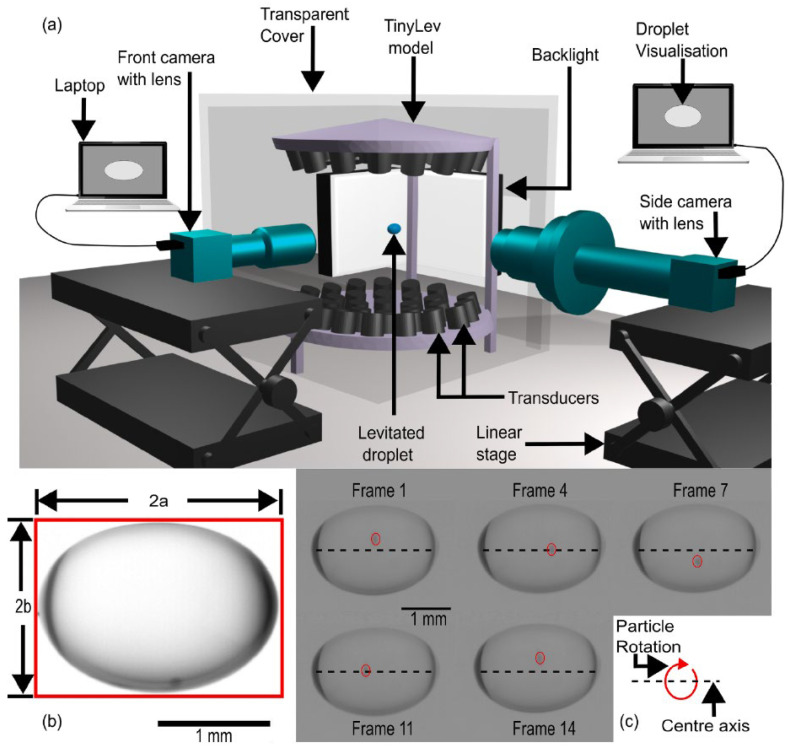
Setup and data collection for measuring the microgravity condition of a droplet levitated in TinyLev. (**a**) Experimental setup including two perpendicular cameras and the acoustic levitator. (**b**) Measurement of droplet dimensions. (**c**) Measurement of rotation speed [7].

Recently, Vashi et al. [7] provided a detailed, theory-based explanation for the existence of a microgravity situation in an acoustically levitated droplet. They used a small-scale, single-axis, non-resonating acoustic levitator, TinyLev, to observe a levitated droplet and describe the microgravity condition (Figure 10). They described the microgravity condition of the droplet as a clinostat by analyzing the rotation of the droplet. This rotation is generated by the ARF that provides a moment due to inevitable misalignment along the levitation axis. The centrifugal acceleration (*a_c_*) of the rotating particle can be approximated using the following equation:

(2) $$\frac{a_{c}}{g}=1.12\;r\;\omega_{rpm}^{2}\times 10^{-3}$$

where *g* is gravitational acceleration, *r* is the radius of the droplet, and ω is the angular velocity of the particle in rpm. Clinostats aim to maintain this centrifugal acceleration below 10^−3^ g to simulate microgravity conditions. To approximate the microgravity situation of levitated droplets, they placed fluorescent microspheres inside the droplets and observed the rotation using front- and side-facing high-speed cameras. Then, they used the observed rate of rotation and the minimum radius to provide the minimum acceleration experience by a particle in the droplet. They took these measurements for 5, 10, and 15 µL droplets and found that increased droplet size reduces angular velocity and thus decreases centrifugal acceleration. From these tests, they found that 15 µL droplets with 7.5 volts supplied to the levitator produced an average minimum centrifugal acceleration of 6 × 10^−2^ g. This value is larger than the 10^−3^ g that is the standard for most on-ground microgravity simulation platforms such as a clinostat, but they concluded that this magnitude shows the potential of the platform to function as a vital microgravity platform. Like the study by Li et al. [5], they also recognized factors such as air perturbations as potentially affecting the stability of the system. They observed that air perturbations cause oscillatory behavior of the droplet which moved the particle away from the center, thus resulting in inconsistent microgravity conditions. Another limitation of TinyLev is the power output limiting the achievable microgravity condition. Ultimately, the capability of scaling up experiments as levitation platforms, particularly with affordable options such as TinyLev, could be limited by the ultrasonic transducers as the ARF limits the mass that can be levitated. Table 1 lists various microgravity simulation platforms as described above.

## 5. Discussion

We reviewed the current state of acoustic levitation and its usages in various studies including biomedical applications. By introducing current on-ground platforms for microgravity, we described the potential of acoustic levitation to be used for microgravity studies. The current research on microgravity simulation applications of acoustic levitation is limited, but studies such as Li et al. [5] and Cao et al. [6] demonstrated that a simulated microgravity environment could exist in acoustically levitated samples. Li et al. [5] examined the biocompatibility of acoustic levitation and discussed possible benefits of an acoustic levitation platform including the open and contactless environment. Cao et al. [6] discussed some potential limitations of the platform including acoustic streaming and convection along with a non-uniform field. Vashi et al. [7] provided the theory-based explanation for a microgravity condition of an acoustically levitated droplet, defining the system as functioning as a microgravity platform analogous to a clinostat due to the rotation of the droplet.

Acoustic levitation has seen many recent advancements and become more accessible with platforms such as TinyLev [4]. However, there is much research that could be conducted to unlock the full potential of acoustic levitation. As we described above, many studies have demonstrated potential uses with biological samples [5,33], such as the viability of acoustically levitated biological samples compared to magnetic levitation. Levitation has shown promise as a platform for artificial cell manipulation [25] and drug processing [72] due to the open, contactless environment. Acoustic levitation can also function as an instrument of manipulation itself, moving samples with ARF. Acoustic levitation also demonstrated its usages in tissue engineering and crystallography applications due to its environment that can allow for unique and repeatable structures to form such as spheroids [33].

While acoustic levitation may currently be viable for use in varying biomedical and biological applications, much remains unresolved for acoustic levitation to be utilized for microgravity study. There exist limitations in acoustic levitation equipment as well as our understanding of the levitating particle behavior. Current research suggests that an acoustically levitated droplet can produce a microgravity condition by functioning as a clinostat [7]. However, the quality of the microgravity condition in a droplet is limited by its volume, which is then affected by the acoustic radiation force. Existing acoustic levitation platforms are currently restricted in their output, so the quality of the microgravity may not reach the standard 10^−3^ g centrifugal acceleration of most clinostats. Additionally, levitated droplets lack the stability of dedicated clinostats. Factors such as air perturbations and acoustic streaming could lead to additional complexities such as inconsistency in particle movement. This may reduce the reliability and measurability of the accelerations experienced by a levitating substance. For acoustic levitation to be a viable microgravity simulator, a more detailed understanding of in-droplet particle rotation and the factors affecting it needs to be reached. With this advanced knowledge, a levitator could be engineered to control the movement of levitating particles, providing stable rotation.

While current levitation platforms are still limited in levitation capacity, advancements in transducer technology will continue to reduce the size and cost while increasing the power capabilities and precise control of ultrasonic transducers [75]. Another way to increase the capacity and control of the levitation environment is by optimizing array geometry [76]. As indicated by Vashi et al. [7], these improvements to droplet capacity in addition to improved understanding and control of droplet behavior could make acoustic levitation platforms available as effective clinostats. Coupled with the open, container-less environment, an acoustic levitator could allow for real-time observation and manipulation of samples within a simulated microgravity environment. Combined, this may open a new research theme and tool based on acoustic levitation for addressing the behavior of biological samples in a microgravity milieu.

## Figures and Tables

**Table 1:** Comparisons of various microgravity simulation platforms.

Platform	Advantages	Disadvantages	References
Clinostat	- Simple design that is easy to make and control - Cheapest option - Well established with many modifications employed	- Mechanical stresses are inevitable and can affect results	[42,43,44,45,46,47,48,49,50,51,52]
Random positioning machine (RPM)	- Microgravity conditions down to 10^-4^ g - Some studies show it to be more accurate than clinostat	- Results can be impacted by mechanical stress due to rotation - Small sample volume	[53,54,55,56,57,58,59,60]
Rotating wall vessel (RWV)	- Larger sample volumes - Readily used as bioreactors - Low shear stress and high mass transfer environment	- High-density cultures sediment, limiting the allowable culture density for reliable microgravity simulation	[61,62,63,64,65,66]
Diamagnetic Levitation	- Unlike rotational platforms, which average the gravity vector to 0, it is balanced at a molecular level - Partial and hypo-gravity environments can be produced	- Samples must be diamagnetic - The necessary strong magnetic fields have been shown to have impacts on cell organization	[67,68,69,70]
Acoustic Levitation	- Affordable and accessible - Open, container-less environment that allows for easy observation and manipulation - Easy to implement with other experimental setups	- Lower-quality microgravity condition than other platforms - Air perturbations and acoustic streaming can limit the consistency of the microgravity environment - Limited sample volume	[5,6,7,25,71,72]

## Data Availability

Not applicable in this review article.

## Abbreviations

The following abbreviations are used in this manuscript:

ARF	Acoustic Radiation Force
RPM	Random positioning machine
RWV	Rotating wall vessels
FG	Function Generator
AMP	Amplifier
HC	High-Speed Camera
ZL	Zoom Lens
MSC	Mesenchymal Stem Cell
SFX	Serial Femtosecond Crystallography
LCP	Liquid-Cubic Phase
ISS	International Space Station
EMF	Electromagnetic Force
YAP	Yes-Associated Protein
STLV	Slow-Turning Lateral Vessel
HARV	High-Aspect Ratio Vessel
